# The Use of Social Media by Healthcare Quality Personnel in Saudi Arabia

**DOI:** 10.1155/2020/1417478

**Published:** 2020-05-22

**Authors:** Turki Alanzi, Doaa Khalid Al-Habib

**Affiliations:** ^1^Health Information Management and Technology Department, College of Public Health, Imam Abdulrahman Bin Faisal University, Dammam, Saudi Arabia; ^2^Royal Commission Hospital, Jubail, Saudi Arabia

## Abstract

**Purpose:**

The objective of this research was to investigate the use of social media for educational purposes by healthcare quality personnel in Saudi Arabia. *Participants and Methods*. A cross-sectional design study was carried out with 78 healthcare quality employees working in different hospitals in Saudi Arabia. The survey was distributed through WhatsApp, and the data were collected during November 2019. The results were analyzed and expressed in percentages using basic statistic tools.

**Results:**

More than half of the participants (74.36%) were under 40 years old, and the gender was equally distributed among them. The participants used the following social media in decreasing order for educational purposes: YouTube, Twitter, LinkedIn, Snapchat, Instagram, and Facebook. The largest proportion of them employed YouTube, and the least used social media network was Facebook. The majority of them (58.87%) employed these platforms more than 3 hours daily. Most respondents (82%) agreed that social media can be used to educate on healthcare quality topics, and YouTube was the preferred platform for this goal. The reasons for using social media for professional purposes were networking (27%), education and professional development (24%), and health promotion (13%). Most of the responses considered that social media networks were somehow helpful and very helpful for improving knowledge about the profession (96.20%), improving creativity (90%), improving decision making (83.33%), improving critical skills (80.77%), and improving problem-solving abilities (79.49%).

**Conclusion:**

The findings showed that a high percentage of the healthcare quality personnel in Saudi Arabia used social media for educational purposes, and the most used platform for this objective was YouTube. The results suggested that social media can be potentially useful to perceive healthcare quality in the Kingdom of Saudi Arabia.

## 1. Introduction

According to the definition of Kaplan et al., social media is “a group of Internet-based applications that build on the ideological and technological foundations of Web 2.0, allow the creation and exchange of user-generated content [1].” Ventola also suggested that “the definition of social media is broad and constantly evolving. The term generally refers to Internet-based tools that allow individuals and communities to gather and communicate; to share information, ideas, personal messages, images, and other contents; and, in some cases, to collaborate with other users in real time [2].”

Globally, social media platforms are useful tools for healthcare professionals, and they can be used for different purposes such as education; professional development; job search; health promotion; personal promotion; communication with patients, colleagues, and students; dissemination of information about health; discussion of public health policies; and analysis of various issues related to general health topics [2]. In this regard, several medical specialties such as pediatrics, radiology, oncology, pathology, cardiology, emergency and critical care, nursing, surgery, dentistry, pharmacy, health care quality, and others have used social media for many of the purposes previously mentioned [3–9].

Also, some specific applications about the use of social media to improve health education in different specialties of the medical field are presented in numerous previous studies [10–26]. For example, one of these studies shows a page published on Facebook about anatomy topics that medical students have considered as an efficient alternative to learning about human anatomy [25]. Another page also published on Facebook has been useful for a group of Thai students interested in understanding the genitourinary system [26]. Likewise, Ghahramani et al. highlighted the interest of medical students and professionals in the use of social networks for education and professional training in cardiology [17]. Also, several specialists in the field of medicine considered that social networks such as Facebook, Twitter, and others are useful for education, thanks to the possibility of sending images such as electrocardiograms, articles, scientific illustrations, videos, audios, and a wide variety of information that contribute to the educational process of medical professionals [23]. In another study conducted in Saudi Arabia, students were found to be comfortable and satisfied when using WhatsApp as a useful tool for learning in a health informatics course [24]. Besides, Ranginwala et al. emphasized the importance of social networks such as Twitter and Instagram in radiological education; the authors described how these tools can be used to send messages on topics of medical interest, research summaries, anatomical images, videos, and other types of information beneficial in radiology education [5].

Furthermore, health professionals have used social media platforms such as Facebook, YouTube, Twitter, WhatsApp, Snapchat, Instagram, LinkedIn, and others to promote learning and improve the theoretical and practical knowledge of the profession, research, publish, and disseminate relevant scientific information [2, 3, 10, 27–33]. For instance, some of these cited studies indicated the importance of social media in knowledge sharing and continuing education; the use of social media in teaching and learning in medical education through participatory models; the possibility of using social media as an open-learning resource in medical education; the importance of social media in clinical research; and the role of doctors in the dissemination of health information [10, 29, 30, 32, 33].

On the other hand, additionally to the benefits provided by social media to communicate, learn, and improve education and medical practice, several authors have warned about the barriers, risks, harms, and ethical problems involved in the inappropriate use of these communication media [2, 27, 30]. In particular, they commented that some of the information published on social media is inaccurate or not well supported scientifically, and believe that sometimes the published information can affect the privacy of patients or doctors. Therefore, some authors think that the students and teachers should critically evaluate the contents available in social media because, sometimes, the published information is not precise or exact [34–36].

Regarding healthcare quality departments whose general mission is to promote safety, quality, and protect patients and health care personnel in hospitals, it was noted that only a few articles on the use of social media have been published in this field [9, 37–41]. In this regard, Ranney et al. remarked that the use of social media in quality healthcare is a promising nascent field [9]. This observation can be seen in the works of Padrez et al. and Hawkins et al. [38, 39]. The first of these studies suggested that connecting social media and medical record data can be useful to observe the quality of healthcare [38]. Similarly, the second study found that patient tweets from several hospitals in the United States could serve to perceive the quality of care of these institutions [39]. However, there are no specific studies on the use of social media for educational purposes by providers of quality healthcare.

Concerning Saudi Arabia, there has been no study related to this subject. But, considering the high penetration rate of social media in this country, it is possible to suggest that there is potential to use social networks to improve the healthcare quality in the Kingdom of Saudi Arabia [42]. Therefore, the objective of this research was to investigate the use of social media for educational purposes by healthcare quality personnel in Saudi Arabia. In particular, this study will contribute to raising awareness of the importance of social media in the educational training of quality healthcare providers in this country.

## 2. Materials and Methods

### 2.1. Study Settings and Participants

A cross-sectional design study was carried out using an online-based questionnaire to know the opinion of the healthcare quality employees on the use of social media as an educational tool in Saudi Arabia. In other words, our research question was to find out if social media can be used as a tool to improve the education of quality healthcare providers in this country. The number of participants was 78 healthcare quality employees working in different hospitals in Saudi Arabia. The completion of the questionnaire was considered to imply informed consent to participate in the study, and the ethical approval was obtained from the Institutional Review Board of the Iman Abdulrahman Bin Faisal University. The ethical approval referring number was IRB-PGS-2019-03-90.

**Figure 1:**
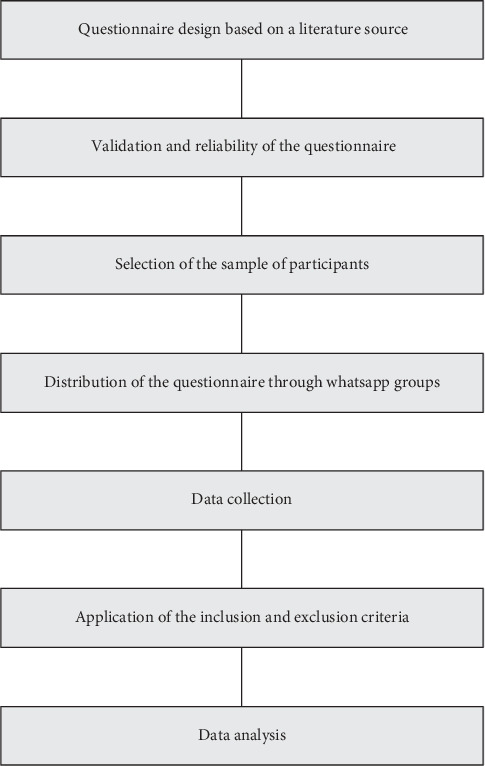
Sequence of the steps used in the methodology procedure (*n* = 78).

Figure 1 shows a diagram of the methodological procedure used in this research. According to this scheme, the main sequential steps of the research survey were as follows: (1) the design of the questionnaire; (2) validation and assessment of the reliability of the questionnaire; (3) selection of the sample of participants; (4) distribution of the questionnaire survey to the participants through WhatsApp groups; (5) data collection; and (6) application of the inclusion and exclusion criteria.

### 2.2. Design and Description of the Questionnaire

The questionnaire of the survey was designed based on a model developed by Alsobayel in a previous study [43]. The only difference between the published questionnaire and the one used in this study was the objective. Alsobayel designed the questionnaire to investigate the use of social media for professional purposes. But, we used the mentioned questionnaire to examine the use of social media for educational purposes by the quality healthcare professional in Saudi Arabia. The experts who examined the questionnaire agreed with the proposed questionnaire. The questionnaire is shown in Appendix.

The survey questionnaire consisted of 10 questions. Most of the questions were multiple choice questions. The first part of the survey contained 4 questions about the demographic information of the participants: age, gender, level of education, and working field. And, the second part of the questionnaire involved 6 questions concerned with the use of social media as an educational tool. The questions of the second part of the survey were related to the type of social media applications used by the participants for education purposes: Facebook, Twitter, Instagram, LinkedIn, YouTube, and Snapchat; daily hours of use of social media; reasons for using social media networks professionally; use of social media networks for improving knowledge about healthcare quality; rating the use of different social media application for education and professional development; and rating the impact of this use on professional development and practice on improving knowledge, critical thinking, problem-solving abilities, creativity, and professional decision making.

### 2.3. Validation and Reliability of the Questionnaire

The questionnaire was validated by 3 experts who agreed with the proposed questionnaire.

Furthermore, the reliability of the questionnaire was verified by carrying out 3 pilot tests with 5 participants each. Participant responses were found to be similar overall. These tests suggested that the questionnaire was valid and reliable.

### 2.4. Selection of the Sample of Participants

**Figure 2:**
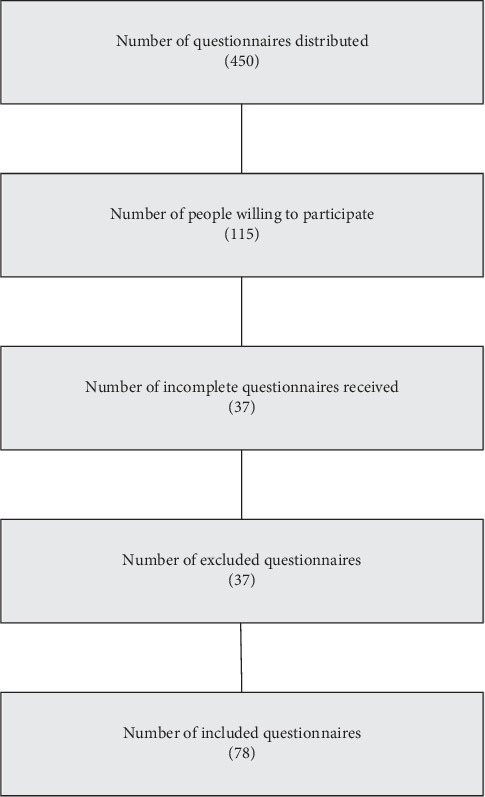
Flowchart of the population (*n* = 78).

The questionnaire was sent to 450 healthcare providers from Saudi Arabia belonging to the WhatsApp groups. However, only a sample of 115 healthcare providers (25.56%) responded to the questionnaire. The flowchart of the population is presented in Figure 2. The figure indicates that the number of distributed questionnaires was 450. Also, 115 quality field healthcare providers were willing to participate. 37 incomplete questionnaires were received and excluded. The number of included questionnaires was 78.

### 2.5. Distribution of the Questionnaire Survey to the Participants

The survey was distributed among the participants through the WhatsApp groups available in Saudi Arabia for healthcare providers. The questionnaire was anonymous.

### 2.6. Data Collection

The data of the survey were collected and analyzed using the online website QuestionPro. The survey distributed among the participants through WhatsApp, and data was collected using WhatsApp platform during November 2019. The questionnaire was available through the following link [44]. The privacy of the participants was kept by storing the data in a codified version on the Unit Server.

### 2.7. Inclusion and Exclusion Criteria

After data collection, we applied the inclusion and exclusion criteria assumed in this study. We only included male and female staff working in the field of quality healthcare in Saudi Arabia. The rest of the participants were excluded. A group of 78 participants met the inclusion criteria. This sample size was small because the number of quality healthcare employees is not very high in most hospitals in Saudi Arabia.

### 2.8. Data Analysis

The survey data were analyzed using basic statistic tools, and the results are presented in tables and figures in terms of percentages relative to the total number of participants. The mean and the standard deviation of the data were calculated using the survey website.

## 3. Results

Table 1 shows the demographic data of the participants, and we can observe that the majority of them (74.36%) were under 40 years old, while the gender was equally distributed among them: male 50% and female 50%. Also, almost half of the respondents (46%) had a bachelor's degree, and the rest of them were diploma, master, and doctorate holders. All the participants were working in the field of quality healthcare.

**Figure 3:**
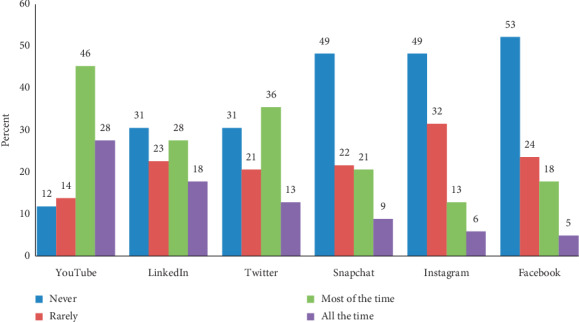
Social media networks used for improving knowledge about healthcare quality (*n* = 78).

Concerning the use of social media for improving knowledge among quality healthcare providers, Figure 3 shows that most of the respondents used YouTube for this purpose. This figure also indicated that the least used social media network for this objective was Facebook. The responses were expressed in terms of never, rarely, most of the time, and at all times.

Table 2 gives us an idea about the time of use of social media by the participants, and it can be observed that the majority of them (58.87%) used these platforms more than 3 hours daily (29.49%).

Regarding the use of social media networks to improve knowledge about healthcare quality, most of the respondents (82%) agreed that social media networks can be used for this purpose.

**Figure 4:**
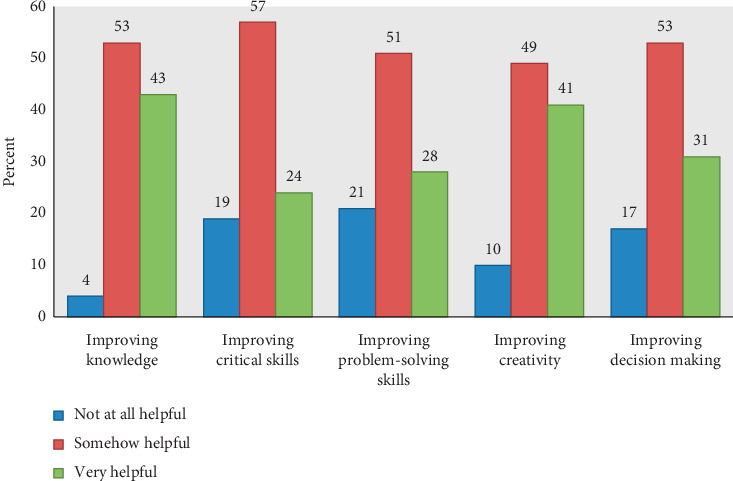
Impact of using social media on education and professional development (*n* = 78).

Alike, in Figure 4, we can observe the impact of using social media networks on education and professional development. The participants rated the impact in terms of very helpful, somehow helpful, and not at all helpful. Most of the responses agreed that all the listed social media were somehow helpful and very helpful for improving knowledge about the profession (96.20%), improving creativity (90%), improving decision making (83.33%), improving critical skills (80.77%), and improving problem-solving abilities (79.49%).

**Figure 5:**
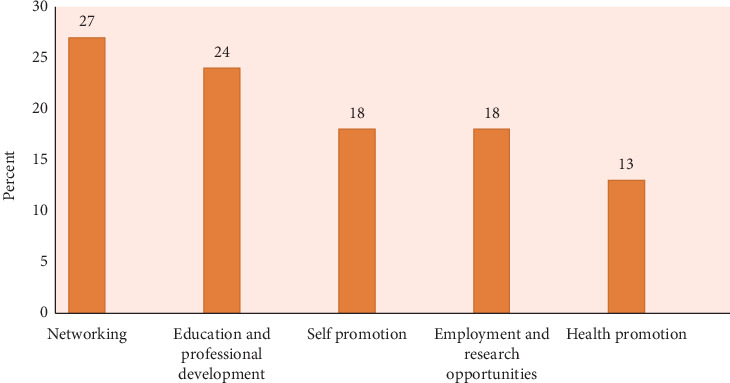
Reasons for using social media for professional purposes (*n* = 78).

The reasons for using social media for professional purposes are shown in Figure 5. Here, we observe that the participants employed social media mostly for networking (27%), education and professional development (24%), and health promotion (13%).

## 4. Discussion

The general findings of this study suggested that the use of social media networks was beneficial for the education of the personnel working in the field of quality healthcare in Saudi Arabia.

Regarding the social media platforms used by the participants for education, Figure 3 shows that the participants utilized the most common social media networks available in Saudi Arabia in the following decreasing order: YouTube, Twitter, LinkedIn, Snapchat, Instagram, and Facebook. The largest proportion of them employed YouTube, and the least used social media network was Facebook. Concerning these results, a study conducted in Saudi Arabia also found that YouTube was the most used social network for the education of medical students [45]. Likewise, in another investigation carried out in the United States, it was observed that the majority of the students of a continuing medical education course used YouTube for educational purposes [46]. On the other hand, it is pertinent to comment that although YouTube was used preferentially in our study for improving knowledge about healthcare quality, some authors have criticized the use of YouTube for educational purposes because the content is not supervised [47].

As indicated in the results section, the majority of participants (82%) pointed out that they used the social media mentioned previously for improving knowledge about healthcare quality. This high percentage suggested that social media networks were widely used by the participants for education in healthcare quality that can be shared and accessed easily through these platforms. Related to this finding, various studies have shown that social media networks can be employed as useful instruments for education in diverse medical specialties such as anatomy, neurology, cardiology, odontology, radiology, oncology, nursing, pharmacy, and other areas of the medical field [5, 11–25]. These networks can be used as a complementary tool to create a suitable environment for education because they contribute to improve the teaching-learning process and help the understanding of theoretical and practical knowledge in medical schools [25, 26, 34, 48, 49]. Also, most of the students feel satisfied and engaged when social media networks and other tools are incorporated as an educational strategy in medical education [26, 48, 50].

On the impact of the use of social media in quality healthcare education, Figure 4 depicts that according to the opinion of the participants, these tools helped improve knowledge about the profession, creativity, professional decision making, critical skills, and problem-solving skills. The development of these abilities is important in any educational process because they contribute to enhancing innovation skills, learning competencies, communication capabilities, academic performance, evaluation judgment, social success, abilities to make good decisions, and so forth [51, 52]. On this subject, in a previous study, it was observed that social media were beneficial for improving knowledge about the profession [43]. In relation to this outcome, it is important to mention that social media networks have been extensively used in medical education, and they can be a useful instrument for this purpose when implemented correctly [53]. On the other hand, it is important to mention that only a relatively low proportion of the participants believed that social media networks were not at all helpful for education purposes.

As can be seen in Figure 5, the first reason why the participants used social media professionally was for networking (26.79%), which is expected since people usually use social media to connect and network for different purposes. The second reason for using these platforms was education and professional development (24.11%), which is the focus of our work. Here, we see that almost a quarter of the healthcare quality personnel employed social media for educational purposes. As a comparison, a similar result was observed in a radiology department in Saudi Arabia where 21.1% of the personnel employed social media for study [54]. It is relevant to highlight that education contributes to the acquisition of knowledge, skills, competencies, abilities, and effective procedures that benefit professional development [55]. Additionally, participants used social media for other reasons such as self-promotion, employment and research opportunities, and health promotion.

In general, our results coincide with the findings of some studies that have also found that social media were useful to improve the theoretical and practical knowledge of the medical profession [25, 26, 34, 48, 49, 56]. For example, Alshakhs et al. found that almost half of healthcare providers felt that social media was useful for improving knowledge about the profession [57]. Also, Almaiman et al. observed that social media improved the knowledge and practice of health professionals [56].

The main limitation of this study was the small sample size of the participants. In this regard, it should be noted that the number of quality health workers is not very high in most hospitals in Saudi Arabia; so, for this reason, we believe the sample size was small. Another limitation of our study was related to the fact that it was not possible to infer the implications of the results on the methodological, theoretical, and practical aspects of health education. Furthermore, we could not frame our research within the framework of any communication theory. Likewise, another deficiency of this investigation was that we did not contextualize this study with the reality of the health sector in Saudi Arabia. In other words, we were unable to deduce how sociocultural and demographic factors may affect the results of this research. Alike, we did not investigate whether there were differences between professionals regarding the hours used, the improvement of knowledge, or the reasons for using social media.

In future studies, we will try to increase the sample size by incorporating people from the majority of the healthcare quality departments of the Saudi Arabian hospitals. Also, we will try to overcome the other mentioned limitations. Furthermore, we will investigate the use of social networks to perceive the quality of medical care in the Kingdom of Saudi Arabia.

Also, it is pertinent to comment that this study has been the first one carried out in Saudi Arabia to find out if social networks were used for educational purposes by quality healthcare employees.

## 5. Conclusion

The findings showed that a high percentage of the healthcare quality personnel in Saudi Arabia used social media networks for improving education and that the most used platform for this purpose was YouTube. The results suggested that social media can be potentially useful to perceive healthcare quality in the Kingdom of Saudi Arabia.

## Figures and Tables

**Table 1:** Demographic data (*n* = 78).

Characteristic	*n*	%
*Age*		
21–30	14	17.95
31–40	44	56.41
41–50	16	20.51
51–70	4	5.13
Mean	2.13	
Standard deviation	0.76	
Variance	0.58	

*Gender*		
Male	39	50
Female	39	50
Mean	1.50	
Standard deviation	0.50	
Variance	0.25	

*Professional qualification*		
Diploma	4	5.13
Bachelor	36	46.15
Master	29	37.18
Doctorate	9	11.54
Mean	2.55	
Standard deviation	0.77	
Variance	0.59	

**Table 2:** Daily social media usage (*n* = 78).

Hours used	*n*	%
Less than an hour	9	11.54
Up to 2 hours	23	29.49
Up to 3 hours	16	20.51
Up to 4 hours	14	17.95
Up to 5 hours	16	20.51
Mean	3.06	
Standard deviation	1.33	
Variance	1.78	

## Data Availability

The data used to support the findings of this study are available from the corresponding author upon request.

## Appendix

Survey questionnaire.

(1) Age (years)
    ( ) 21–30
    ( ) 31–40
    ( ) 41–50
    ( ) 51–70
(2) Gender
    ( ) Male
    ( ) Female
(3) Nationality
(4) Professional qualification
    ( ) Diploma
    ( ) Bachelor
    ( ) Master
    ( ) Doctorate
(5) Working in healthcare quality field
    ( ) Yes
    ( ) No
(6) Which of the following social media networks do you use? Select one or more
    ( ) Facebook
    ( ) Twitter
    ( ) Instagram
    ( ) LinkedIn
    ( ) YouTube
    ( ) Snapchat
(7) How much time do you usually spend using social media networks on daily basis?
    ( ) Less than 1 hour
    ( ) Up to 2 hours
    ( ) Up to 3 hours
    ( ) Up to 4 hours
    ( ) Up to 5 hours
(8) What are the reasons for using social media networks professionally? Choose one or more
    ( ) Networking
    ( ) Health promotion
    ( ) Employment and research opportunities
    ( ) Education and professional development
    ( ) Self-promotion
(9) Rate the impact of using social media networks on your professional development and practice.
(10) Rate the impact of using social media networks on your professional development and practice.
    Improving knowledge about your profession
     ( ) Not at all helpful
     ( ) Somehow helpful
     ( ) Very helpful
    Improving critical skills
     ( ) Not at all helpful
     ( ) Somehow helpful
     ( ) Very helpful
    Improving problem-solving skills
     ( ) Not at all helpful
     ( ) Somehow helpful
     ( ) Very helpful
    Improving creativity
     ( ) Not at all helpful
     ( ) Somehow helpful
     ( ) Very helpful
    Improving professional decision making
     ( ) Not at all helpful
     ( ) Somehow helpful
     ( ) Very helpful
